# Regarding the role of systemic immune-inflammation index on the risk of chronic kidney disease

**DOI:** 10.1097/JS9.0000000000001097

**Published:** 2024-01-18

**Authors:** Wei Yang, Jinlin Liu

**Affiliations:** a Department of Clinical Laboratory, Zhejiang Provincial People’s Hospital Bijie Hospital, Bijie, Guizhou; b Department of Clinical Laboratory, South China Hospital, Medical School, Shenzhen University, Shenzhen, People’s Republic of China

*Dear Editor*,

We read Zhao’s study^1^ with great interest. In this study, the author aims to investigate the relationship between systemic immune-inflammation index (SII) and chronic kidney disease (CKD), while the SII is a novel index based on the lymphocyte, neutrophil, and platelet counts. The author mentioned that more and more evidence reveals that the SII is a useful index reflecting the systemic immune and inflammatory status of the human body^2^. Despite definite results, as laboratory technicians, we raise some concerns about some factors that could greatly influence these laboratory parameters in this study.

A very important confounder may also be ignored when they depict the results of univariate and multivariable analyses of SII with CKD, ACR (albumin-to-creatinine ratio), and eGFR (estimated glomerular filtration rate), respectively, that is the commonly used drugs for the treatment of CKD. Take glucocorticoid, for example, lymphocyte, neutrophil, and platelet counts were all greatly influenced by the glucocorticoid. However, the drug use history or their statistical significance in Zhao’s study were not listed. Moreover, not only 6815 CKD patients were documented in this study; other 7345 diabetes patients and 16 682 hypertension patients were also included in this study, while these patients may have also received other drugs, which could also influence the SII results. Thus, the statistical significance of univariate and multivariable analyses of SII could result in inappropriate results in Khalid’s study. Moreover, the previous cohort did not receive any prior treatments^2^, while the author mentioned that SII is a useful index reflecting the systemic immune and inflammatory status of the human body. Thus, to minimize the drug influence on the statistical results and validate the role of SII on CKD, the author could use another validation cohort to validate their results.

However, although we felt the need to address this point, we congratulate Zhao’s outstanding work^1^, which gives us this opportunity to discuss the relationship between CKD and SII.

## Ethical approval

Not applicable.

## Consent

Not applicable.

## Sources of funding

Not applicable.

## Author contribution

W.Y. and J.L.: discussed and wrote the manuscript.

## Conflicts of interest disclosure

We declare no conflicts of interest.

## Research registration unique identifying number (UIN)

Not applicable.

## Guarantor

Jinlin Liu.

## Provenance and peer review

Not applicable.

## Data availability statement

This is a comment on a published paper; no data was presented in our comment, so the data statement is not applicable.

## References

[1] Zhao X Wang T Zhou L. Dose–response analysis of systemic immune-inflammation index and risk of chronic kidney disease. Int J Surg 2023. [Epub ahead of print]. 10.1097/JS9.0000000000001007 PMC10942233 38116671

[2] Hu B Yang XR Xu Y. Systemic immune-inflammation index predicts prognosis of patients after curative resection for hepatocellular carcinoma. Clin Cancer Res 2014;20:6212–6222. 25271081 10.1158/1078-0432.CCR-14-0442

